# Starch-Assisted Eco-Friendly Synthesis of ZnO Nanoparticles: Enhanced Photocatalytic, Supercapacitive, and UV-Driven Antioxidant Properties with Low Cytotoxic Effects

**DOI:** 10.3390/ijms26020859

**Published:** 2025-01-20

**Authors:** Roumaissa Djafarou, Ouarda Brahmia, Soumia Haya, Ertugrul Sahmetlioglu, Fatma Kılıç Dokan, Tarek Hidouri

**Affiliations:** 1 Laboratoire des Techniques Innovantes de Préservation de l’Environnement, Université de Constantine 1, Constantine 25000, Algeria; roumaissa.djafarou@student.umc.edu.dz (R.D.); ouarda.brahmia@umc.edu.dz (O.B.); 2 Département de Physique, Université de Constantine 1, Constantine 25000, Algeria; hayasoumia13@gmail.com; 3 Department of Basic Sciences of Engineering, Kayseri University, Kayseri 38039, Turkey; sahmetlioglu@kayseri.edu.tr; 4 Department of Chemistry and Chemical Processing Technologies, Mustafa Çıkrıkcıoglu Vocational School, Kayseri University, Kayseri 38039, Turkey; fatmakilic@kayseri.edu.tr; 5 Department of Mathematical, Physical and Computer Sciences, University of Parma, 43124 Parma, Italy

**Keywords:** ZnO semiconductor, nanoparticles, green synthesis, starch biotemplate, sol-gel method, photocatalysis, supercapacitors, UV-induced antiradical activity, environmental remediation, cytotoxicity

## Abstract

This study presents an efficient and environmentally sustainable synthesis of ZnO nanoparticles using a starch-mediated sol-gel approach. This method yields crystalline mesoporous ZnO NPs with a hexagonal wurtzite structure. The synthesized nanoparticles demonstrated remarkable multifunctionality across three critical applications. In photocatalysis, the ZnO NPs exhibited exceptional efficiency, achieving complete degradation of methylene blue within 15 min at pH 11, significantly surpassing the performance of commercial ZnO. Under neutral pH conditions, the nanoparticles effectively degraded various organic dyes, including methylene blue, rhodamine B, and methyl orange, following pseudo-first-order kinetics. The methylene blue degradation process was aligned with the Langmuir–Hinshelwood model, emphasizing their advanced catalytic properties. For supercapacitor applications, the ZnO NPs attained a high specific capacitance of 550 F/g at 1 A/g, underscoring their potential as energy storage solutions. Additionally, the nanoparticles demonstrated strong UV-induced antiradical activity, with an EC_50_ of 32.2 μg/mL in DPPH assays. Notably, the cytotoxicity evaluation revealed an LC_50_ of 1648 μg/mL, indicating excellent biocompatibility. This study highlights a sustainable approach for the synthesis of multifunctional ZnO NPs that offers effective solutions for environmental remediation, energy storage, and biomedical applications.

## 1. Introduction

Zinc oxide nanoparticles (ZnO NPs) have garnered significant attention in materials science due to their versatile applications in photocatalysis [1,2], energy storage [3,4], and environmental remediation [5,6]. The unique semiconducting and surface properties of ZnO, including its wide bandgap (3.37 eV) and high exciton binding energy (60 meV) [7], make it an ideal candidate for addressing contemporary challenges in clean energy production [8], water purification [9], and advanced energy storage technologies [10]. While conventional synthesis methods for ZnO nanoparticles have been extensively studied, there is a growing emphasis on green synthesis approaches that minimize environmental impact, while enhancing the material’s functional properties [11]. These eco-friendly methods not only reduce the use of harmful chemicals but also frequently result in nanoparticles with improved biocompatibility and reduced toxicity [12,13]. This study investigated the sol-gel synthesis of ZnO nanoparticles using starch as a biotemplate and stabilizing agent with the objective of developing a multifunctional nanomaterial with enhanced photocatalytic, supercapacitive, and UV-induced antiradical properties. The sol-gel method offers precise control over particle size, morphology, and crystallinity, enabling the production of highly uniform nanoparticles at the nanoscale [14]. Incorporating starch as a bio-derived stabilizing agent is expected to narrow the nanoparticle dispersion range and improve their stability. Starch, a natural biopolymer, can form protective layers around nanoparticles, potentially enhancing their colloidal stability and preventing early agglomeration [15]. It is hypothesized that this eco-friendly synthesis approach will yield ZnO nanoparticles with superior performance characteristics compared to those produced by traditional methods. The objectives of this research were (1) to elucidate the influence of starch on the structural, morphological, and optical properties of ZnO nanoparticles; (2) to evaluate their photocatalytic performance in the degradation of organic dyes; (3) to assess their electrochemical behavior as supercapacitor electrode materials; (4) to investigate their UV-induced antiradical activity; and (5) to assess their toxicity. Ultimately, this study aims to explore the multifaceted potential of ZnO nanoparticles, specifically their ability to degrade recalcitrant organic pollutants [16], capacity for high-performance energy storage [17], and UV-induced antiradical properties [18]. Through comprehensive characterization techniques, including X-ray diffraction (XRD), scanning electron microscopy (SEM), infrared (IR) spectroscopy, and UV-visible spectroscopy, this study sought to provide insights into the structure–property relationships that govern the multifunctionality of these ZnO nanoparticles. A Brunauer–Emmett–Teller (BET) investigation brought further information on the texture and surface area of the nanoparticles.

## 2. Results and Discussion

### 2.1. Characterization of ZnO NPs

#### 2.1.1. UV-Visible and IR Analysis

**Figure 1:**
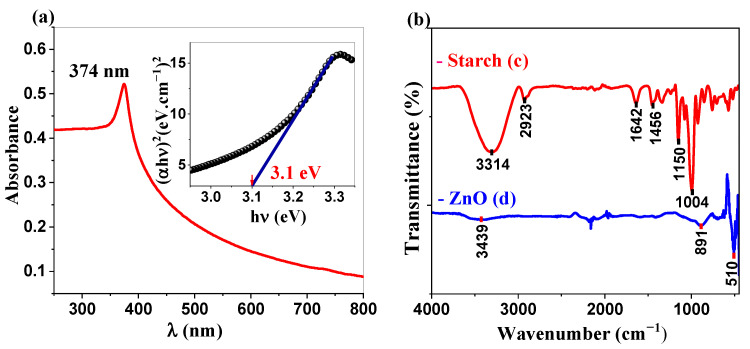
(**a**) UV-visible spectrum of ZnO NPs (inset: Tauc plot derived from the UV-visible spectrum). (**b**) FTIR spectra of (c) starch and (d) the synthesized ZnO NPs.

The optical properties of the synthesized ZnO NPs were characterized using UV-visible spectroscopy (Figure 1a). The spectrum exhibited a well-defined excitonic absorption peak at 374 nm, corresponding to the electronic transition from the valence band to the conduction band, consistent with the reported ZnO excitonic absorption values in the range of 350–380 nm [19]. The optical bandgap energy (Eg), determined using the Tauc plot method, where (αhν)^2^ versus photon energy (hν) was plotted and extrapolated to the x-axis (inset, Figure 1a), yielded a value of 3.11 eV [20]. This value aligns with the literature for biosynthesized ZnO nanoparticles of comparable dimensions [21]. This band gap facilitates efficient UV light absorption and charge transfer processes, promoting the generation of reactive oxygen species (ROS), rendering these nanoparticles as potential candidates for environmental applications. In addition to optical properties, FTIR spectroscopy was used to investigate the structural characteristics of the samples. The infrared spectra of the starch and ZnO NPs are presented in Figure 1b (spectra c and d, respectively). The starch spectrum exhibited characteristic absorption bands at 3314 cm^−1^, 2923 cm^−1^, 1642 cm^−1^, and 1004 cm^−1^, corresponding to O-H stretching, C-H stretching, O-H bending of adsorbed water, and C-O stretching [22], respectively, indicating an α-glucosidic structure [23,24]. The FTIR spectrum of the ZnO NPs demonstrated distinctive absorption bands, as shown in Figure 1b (spectrum d). The characteristic absorption band at 510 cm^−1^ corresponds to the Zn-O stretching vibration, confirming the formation of the ZnO wurtzite structure [25,26]. The weak band observed at 3439 cm^−1^ is attributed to O-H stretching vibrations, likely originating from surface-adsorbed moisture or surface hydroxyl groups on the ZnO nanoparticles [27]. This observation was consistent with the complete removal of the starch template during calcination. Furthermore, a minor band at 891 cm^−1^, associated with C-O-C glycosidic bonds [27], indicates minimal residual organic matter, which aligns with the high-temperature calcination process. The FTIR results corroborate the successful synthesis of high-purity ZnO NPs with negligible organic contamination.

#### 2.1.2. Crystallographic Structure and Surface Area Analysis

**Figure 2:**
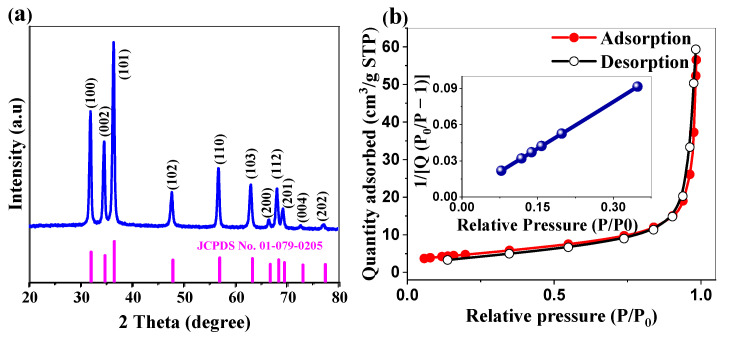
(**a**) XRD pattern of ZnO NPs (in blue) compared with the standard JCPDS card no. 01-079-0205 (in magenta). (**b**) N₂ adsorption–desorption isotherm for BET surface area analysis (inset: BET plot).

XRD analysis was conducted to evaluate the crystalline structure and phase purity of the synthesized ZnO nanoparticles (Figure 2a). The XRD pattern exhibited sharp, well-defined peaks characteristic of the hexagonal wurtzite structure of ZnO, which is consistent with the JCPDS card no. 01-079-0205 [28]. The most intense reflections at 2θ angles of 31.93°, 34.57°, and 36.41° corresponded to the (100), (002), and (101) planes, respectively, confirming the formation of highly crystalline ZnO. Additional peaks at 47.67°, 56.72°, 63.03°, 66.58°, 68.08°, 69.32°, 72.75°, and 77.17° corresponded to the (102), (110), (103), (112), (201), (004), and (202) planes, respectively, indicating the polycrystalline nature of the sample.

Table 1 presents the structural parameters obtained from XRD analysis. The lattice constants a and c were calculated from the XRD data and determined to be 3.23 ± 0.02 Å and 5.18 ± 0.02 Å, respectively, which are consistent with the standard values for the hexagonal wurtzite structure of ZnO [28].

The crystallite size (D) was calculated using the Debye–Scherrer Equation (1):

(1) $$D=\frac{K\times \lambda }{\beta \times cos\theta }$$

where K is the shape factor (0.9), λ is the X-ray wavelength (1.5406 Å for Cu Kα radiation), β is the full width at half maximum (FWHM) in radians, and θ is the Bragg angle [29]. The average crystallite size derived from the three most intense peaks ((100), (002), and (101)) was approximately 22 nm. The narrow peak width and high intensity indicate superior crystallinity, and the absence of extraneous peaks confirms the presence of phase-pure ZnO without detectable impurities or secondary phases.

The textural properties of the ZnO NPs were investigated using N_2_ (g) adsorption experiment at 77 K (Figure 2b). The isotherm exhibits a characteristic Type IV profile according to IUPAC classification, featuring a distinct H3-type hysteresis loop that extends over a broad relative pressure (P/P_0_) range of 0.4–1.0. This H3-type hysteresis, which is characteristic of aggregates of plate-like particles forming slit-shaped pores, supports the predominantly mesoporous nature of the material [30]. The material exhibits a BET-specific surface area of 16.77 ± 0.03 m^2^/g, with a close to 1 coefficient of determination (R^2^ = 0.999) and C value of 171.3 (inset of Figure 2b), indicating a highly reliable surface area determination and strong adsorbate–adsorbent interactions, respectively. The total pore volume of 0.0809 cm^3^/g (determined at P/P₀ = 0.9829) complements BJH analysis results, which indicate uniform pore widths of 23.47 nm and 23.52 nm from adsorption and desorption branches, respectively. The high degree of concordance between these values (Δ = 0.05 nm) confirmed the presence of well-defined, uniform mesopores throughout the material. This well-developed mesoporous architecture confers multiple functional advantages: it facilitates efficient mass transport and maximizes the accessibility of active sites for catalytic reactions [31]; whereas, the interconnected channels enhance light utilization through increased penetration and multiple scattering effects [31]. In energy storage applications, these ordered mesoporous networks create optimal pathways for electrolyte diffusion and ion transport, resulting in enhanced charge storage capacity and superior cycling stability [32]. The combination of crystalline ZnO nanostructures with this precisely engineered porosity provides a versatile platform that is particularly suited for both photocatalytic and electrochemical applications.

#### 2.1.3. Morphological and Elemental Characterization of ZnO Nanoparticles

**Figure 3:**
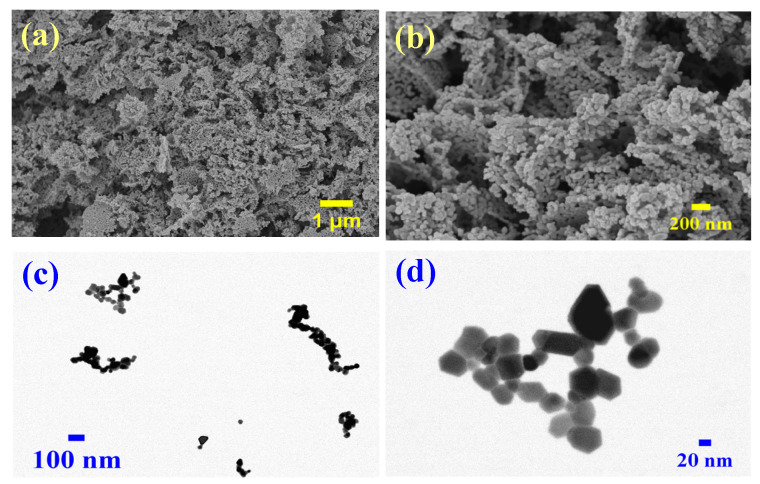
(**a**,**b**) FESEM and (**c**,**d**) STEM images of ZnO nanoparticles.

The structural and morphological characteristics of the synthesized ZnO NPs were investigated by field emission scanning electron microscopy (FESEM) and scanning transmission electron microscopy (STEM). FESEM images (Figure 3a,b) revealed a hierarchical structure with interconnected nanoparticles forming a mesoporous network, directly validating the Type IV isotherm characteristics observed in the BET analysis. The textured architecture exhibits pronounced surface roughness, providing efficient pathways for mass transport and charge transfer [33]. STEM analysis (Figure 3c,d) revealed particles with well-defined boundaries and morphologies ranging from near-spherical to quasi-hexagonal features, characteristic of the wurtzite structure confirmed by XRD analysis [34]. The interparticle network observed through microscopy complements the measured BJH pore width distribution (23.47–23.52 nm), demonstrating the material’s optimized interfacial properties for both photocatalytic and electrochemical applications [35].

**Figure 4:**
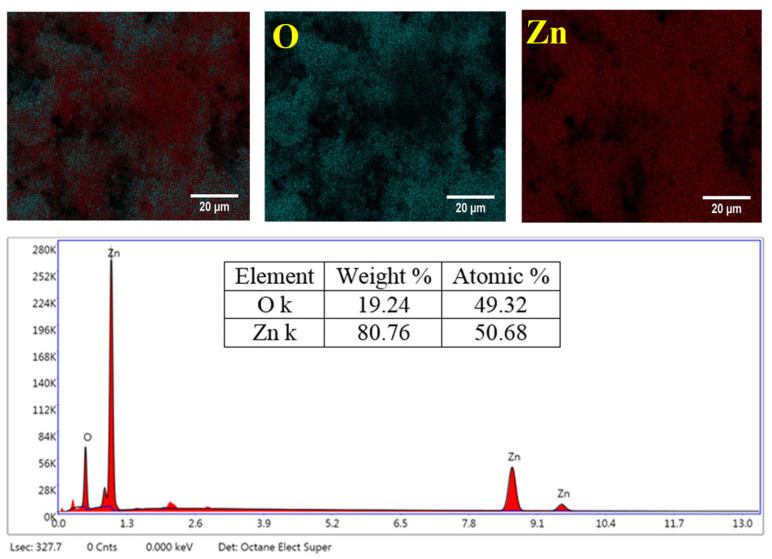
EDS spectrum and elemental distribution mapping of ZnO NPs.

The elemental mapping and EDS spectrum (Figure 4) conclusively demonstrated the chemical purity and compositional uniformity of the biosynthesized ZnO NPs. The mapping results revealed a homogeneous distribution of Zn and O across the analyzed area, emphasizing the uniformity of the material. The quantitative analysis derived from the spectrum indicates an atomic ratio of Zn:O of 50.68:49.32, closely approximating the stoichiometric ratio of ZnO, thereby confirming the successful synthesis of stoichiometric ZnO-NPs. Moreover, the absence of detectable carbon in the EDX analysis corroborated the FTIR results, which showed only trace levels of residual organic species below the detection limit. These observations underscore the efficacy of the green synthesis method for producing high-purity ZnO NPs.

### 2.2. Formation Mechanism of ZnO Nanoparticles

In a similar synthesis method, ref. [27] provides evidence that the formation of zinc oxide ZnO NPs through starch-mediated synthesis represents a sophisticated and environmentally sustainable approach that utilizes the unique dual functionality of starch as both a stabilizing agent and morphology-directing template. The mechanism commences with the dissociation of zinc nitrate (Zn(NO_3_)_2_) in aqueous solution, generating Zn^2+^ ions that are expected to form coordination complexes with the hydroxyl (-OH) groups present in starch molecules [36]. These critical coordination interactions are reported to effectively stabilize Zn^2+^ ions within the starch matrix, preventing premature agglomeration and ensuring controlled particle formation [37]. The synthesis proceeds under mild conditions at natural pH (5.3), maintaining the stability of Zn^2+^ species throughout the reaction medium. According to Kržišnik et al. [38], Zn^2+^ ions remained stable at pH 5.3, as evidenced by their complete removal from aqueous solutions using zero-valent iron nanoparticles (nZVI). This indicates that at this mildly acidic pH, Zn^2+^ exists primarily in its ionic form without significant precipitation as zinc hydroxide (Zn(OH)_2_), which typically occurs at higher pH levels [39]. It is stated that, upon heating to 80 °C, starch undergoes a critical gelatinization process, establishing a complex three-dimensional network that serves as a precise template for controlled nucleation and the growth of ZnO NPs [27]. The authors further affirm that the subsequent calcination at 500 °C initiated the systematic decomposition of the Zn–starch complex, facilitating the formation of well-defined ZnO crystallites through the controlled release of organic residues and subsequent oxidation [27]. The resultant ZnO NPs exhibited a characteristic hexagonal wurtzite crystal structure, as confirmed by XRD analysis, with an optimized average crystallite size of 22 nm (consistent with previous investigations [27,40] and a specific surface area of 16.77 m^2^/g. This innovative green synthesis approach yielded ZnO NPs with precisely tailored properties, enabling their application in diverse fields. These applications include environmental remediation, antimicrobial applications, UV-blocking materials, and advanced energy storage systems, such as high-performance supercapacitors [41].

### 2.3. Photocatalytic Study

The use of metal oxide nanoparticles, particularly ZnO, for photocatalytic degradation of organic pollutants has emerged as a promising strategy in water treatment technologies. This study aimed to comprehensively evaluate the photocatalytic efficiency of biosynthesized ZnO nanoparticles against three model dyes, elucidate the degradation kinetics and underlying mechanisms, and optimize the process parameters to enhance the overall efficiency.

#### 2.3.1. Comparative Dye Degradation

**Figure 5:**
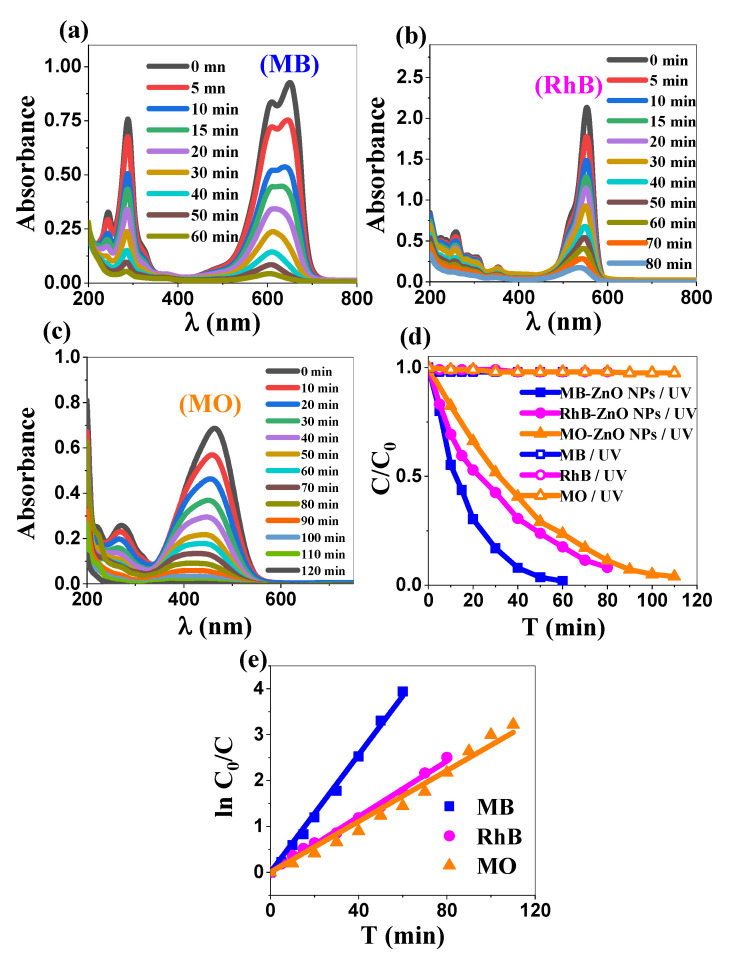
(**a**–**c**) Evolution of the UV-visible spectra of the three dyes MB, RhB, and MO, respectively; (**d**) photocatalytic degradation of the three dyes in the presence and absence of ZnO NPs; and (**e**) linear variation of ln(C₀/C) vs. time.

The photocatalytic activity of the biosynthesized ZnO NPs was systematically evaluated using three model organic dyes, MB, RhB, and MO. Control experiments conducted under UV irradiation in the absence of ZnO NPs confirmed the negligible direct photolysis of the dyes (Figure 5d), evidencing the essential catalytic role of ZnO NPs in the degradation process. To ensure a valid comparison, all initial experiments were conducted under the natural pH conditions of the dye solutions, providing a consistent baseline for subsequent optimization studies. The initial pH values of MB, RhB, and MO were 7.36, 7.72, and 7.78, respectively.

The photocatalytic degradation of MB is evidenced by the gradual decrease in the intensity of its characteristic absorption band, which peaks at 652 nm (Figure 5a). The MB residual concentration at time t was deduced from the measured absorbance of the solution at this wavelength. Similarly, rhodamine B and methyl orange (Figure 5b,c) exhibit a continuous reduction in their respective absorption bands during the course of photocatalysis, indicating their decomposition into smaller compounds [42,43]. It is worth evoking that the breakdown of the dye molecule necessarily generates some intermediate products, which are expected to be in turn photocatalytically degraded into increasingly simpler, environmentally benign compounds. The reaction progresses until potentially complete mineralization is reached [44,45].

As shown in (Figure 5d), the time-dependent degradation profiles under UV irradiation exhibit an exponential decay in the normalized dye concentrations (C/C_0_), with nearly complete degradation achieved within 60, 80, and 110 min for MB, RhB, and MO, respectively. Kinetic analysis revealed that the degradation followed pseudo-first-order kinetics, with apparent rate constants (k_app_) of 0.064, 0.030, and 0.0277 min^−1^ for MB, RhB, and MO, respectively (R^2^ > 0.998). These constants were derived from the linear variation of ln(C_0_/C) versus time (Figure 5e), providing a quantitative measure of the degradation rates and enabling a direct comparison with literature values. The photocatalytic degradation efficiency of the biosynthesized ZnO NPs followed the order MB > RhB > MO.

The observed trend in degradation can be explained by multiple factors. The primary factor is the pH-dependent electrostatic interactions between the charged dye molecules and ZnO surface, which should affect the photocatalytic efficiency, through either adsorption enforcement or its weakening. The point of zero charge (pH_P_zc) of ZnO was determined to be 7.2 (Figure S1), consistent with previously reported ZnO (7.16) biosynthesized using Eucalyptus grandis extract [46]. The initial pH of the solutions (falling within the range 7.3–7.7, for all three dyes) confers a slightly negative surface charge onto the ZnO NPs, hence facilitating the adsorption of cationic dyes (MB and RhB), while inhibiting the adsorption of anionic dyes (MO). Other factors could potentially contribute to the enhanced photocatalytic performance of cationic dyes. MB’s superior degradation stems from its simpler molecular structure compared to RhB and MO [47,48]. Additionally, the energetic alignment between the molecular orbitals (HOMO-LUMO levels) of MB and RhB with the electronic band structure of ZnO enables efficient electron transfer and enhances degradation kinetics [49]. This is exemplified by MB’s complete degradation under optimized conditions [50,51]. The intrinsic properties of the catalyst, including particle size distribution, textural characteristics, and surface properties, also influence photocatalytic performance [52,53]. Furthermore, the molecular stability hierarchy affects the degradation patterns, with simpler structured organic dyes showing higher susceptibility to photocatalytic degradation than the stable azo dye, MO [49]. This analysis revealed that the observed photocatalytic selectivity results from the combined effects of electrostatic interactions with cationic dyes, molecular architecture, electronic structure alignment, and ZnO surface properties. In summary, the degradation order (MB > RhB > MO) correlates with the electrostatic principles governed by the surface charge of ZnO at the operating pH; whereas, MO’s lower degradation rate reflects both electrostatic repulsion and its stable azo structure.

#### 2.3.2. Enhanced Photocatalytic Performance Under Alkaline Conditions and Comparative Analysis

**Figure 6:**
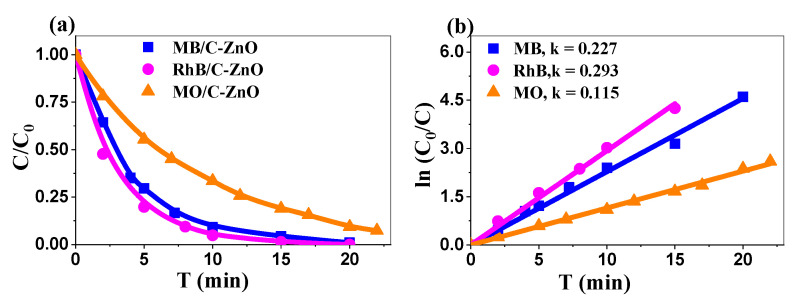
(**a**) Photocatalytic degradation of the three dyes using C-ZnO and (**b**) linear variation of ln(C₀/C) vs. time.

A comparative study revealed that commercial ZnO (C-ZnO) demonstrated significantly higher degradation rates for MB, RhB, and MO dyes at a concentration of 10 ppm under natural pH conditions (Figure 6a). As illustrated in Figure 6b, C-ZnO exhibited apparent rate constants (k_app_) of 0.227, 0.293, and 0.115 min^−1^ for MB, RhB, and MO, respectively. The k_app_ values for both the commercial and biosynthesized ZnO under natural pH conditions are listed in Table 2.

**Figure 7:**
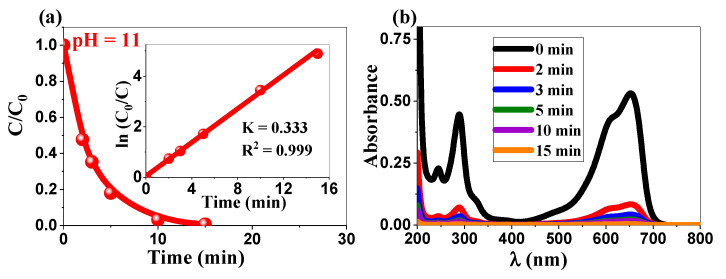
(**a**) Photocatalytic degradation of MB (pH 11) using ZnO NPs (inset (**a**) linear variation of ln(C₀/C) vs. time) and (**b**) UV-visible spectrum evolution of the MB dye.

Despite the notable specific surface area of the biosynthesized ZnO NPs, the superior photocatalytic performance of commercial ZnO can be attributed to its controlled production processes that ensure consistent physicochemical properties and higher purity [54], as well as optimized structural and electronic properties for efficient light absorption and pollutant interactions [55]. Where biosynthesized ZnO may exhibit variations in particle size, morphology, and surface properties, commercial ZnO maintains more uniform characteristics that contribute to consistent photocatalytic activity [56]. Nevertheless, significant advancement was achieved in this work when MB was subjected to optimization studies, particularly under alkaline conditions (pH 11) (Figure 7).

The significant enhancement in the photocatalytic activity of ZnO NPs for MB degradation at pH 11 can be attributed to a few interconnected factors. At the higher end of the pH scale, the surface of the ZnO NPs acquired an extensive negative charge, substantially increasing the amount of adsorbed cationic MB molecules [57,58]. In turn, the higher number of molecules in close contact with the surface facilitates electron transfer and induces more efficient degradation. Furthermore, an alkaline environment promotes the generation of hydroxyl radicals (^•^OH), which act as the primary reactive species responsible for the photocatalytic degradation of organic pollutants [59]. The superior performance under alkaline conditions was evidenced by the substantial increase in the pseudo-first-order rate constant k_app_ to 0.33 min^−1^ (inset a, Figure 7) significantly exceeding both the neutral pH performance (0.064 min^−1^) and that of commercial ZnO (0.227 min^−1^ at neutral pH). Thus, in summary, this enhancement is attributed to two key mechanisms: the increased concentration of hydroxyl ions reacting with photogenerated holes to form hydroxyl radicals (^•^OH) [60] and the optimized electrostatic interactions between the negatively charged ZnO surface and cationic MB molecules [61]. The significant continuous reduction in the MB absorption spectrum (Figure 7b) allows for the visualization of the enhanced degradation efficiency under alkaline conditions. Supporting our results, some studies have found that the photocatalytic activity of ZnO NPs can be pH dependent and may vary for different dyes. For instance, Alharthi et al. [47] reported that ZnO NPs exhibit more favorable behavior for the adsorption and photocatalytic degradation of methyl orange (MO) compared to MB. This suggests that the optimal pH for degradation varies depending on the specific dye and properties of the biosynthesized ZnO NPs. In essence, our findings reveal that moderately alkaline conditions (pH 11) optimize the performance of ZnO NPs for MB degradation, yielding a rate constant that is five times higher than that at neutral pH. This notable enhancement establishes the critical need to adjust the pH to a value that maximizes the photocatalytic activity of biosynthesized ZnO NPs for water remediation.

In comparison to literature data, the photocatalytic performance of the biosynthesized ZnO NPs was significantly higher than those of various reported ZnO-based systems (Table 3). For MB degradation, the catalyst showed an important performance at pH 11 with a notably high apparent rate constant (k_app_ = 0.33 min^−1^), resulting in complete degradation in a short period of 15 min. This performance surpasses that of several modified ZnO systems, including 2% Fe-ZnO (k_app_ = 0.0107 min⁻¹) [62], Cu₂O/ZnO/GO composites [63], and NiO-ZnO (k_app_ = 0.015 min^−1^) [60]. Even at a neutral pH (7.36), the ZnO NPs maintained substantial activity (k_app_ = 0.064 min^−1^), demonstrating comparable efficiency to Cu-ZnO systems (k_app_ = 0.137 min^−1^ at 45 °C) [64]. For RhB degradation, the biosynthesized ZnO NPs exhibited a competitive k_app_ value of 0.03 min^−1^ with a 92% degradation rate in 80 min, closely matching that of ZnO NPs synthesized via solution phase methods (k_app_ = 0.034 min^−1^, 95% degradation) [65] and exceeding that of Se-ZnO NPs (k_app_ = 0.014 min^−1^) [66]. For MO degradation, the biosynthesized ZnO NPs displayed activity (k_app_ = 0.027 min^−1^, 96% degradation), which is superior to its analogs synthesized through conventional or modified approaches. Thus, it surpasses the performance of ZnO NPs (k_app_ = 0.00417 min^−1^) [67] and 1% Ce-ZnO NPs (k_app_ = 0.015 min^−1^) [67], both of which were prepared using a laser-assisted chemical bath method. The enhanced photocatalytic activity can be attributed to the distinctive inherent physicochemical properties of the biosynthesized ZnO NPs. Their specific surface area (16.77 m^2^/g) and mesoporous architecture create strategically distributed adsorption sites and facilitate efficient mass transfer. These textural advantages contribute to the superior photocatalytic performance observed for single-dye systems.

#### 2.3.3. The Impact of Isopropanol on Photocatalytic Dye Degradation Rate: Mechanistic Insights

**Figure 8:**
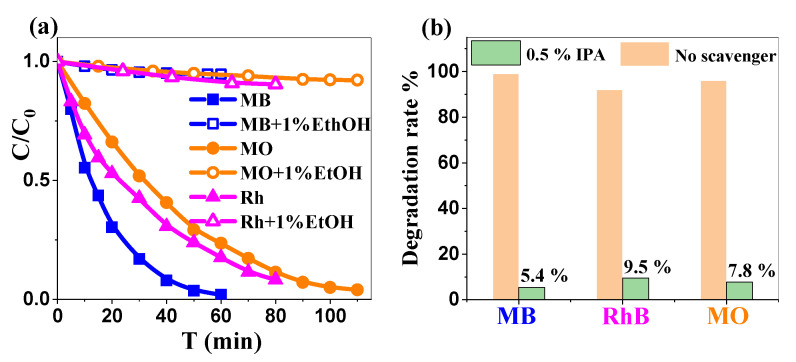
(**a**) The photocatalytic degradation of organic dyes by ZnO NPs in the presence of isopropanol (0.5%) vs. its absence. (**b**) Degradation rate % of the dyes in the presence and absence of IPA, respectively.

The addition of isopropanol (IPA), which is known to act as a hydroxyl radical (^•^OH) scavenger [68], provided significant insights into the photocatalytic degradation mechanism of organic dyes by ZnO NPs. Under neutral pH conditions, the degradation of MB, RhB, and MO (10 ppm each) followed pseudo-first-order kinetics in the absence of IPA. The addition of 0.5% IPA to each system decreased the degradation efficiency by a substantial 90–95% (Figure 8a), confirming the expected predominant role of ^•^OH radicals in the photocatalytic process [69].

This significant inhibition may be chiefly attributed to three mechanisms that act in conjunction. The primary inhibition mechanism involves IPA directly capturing ^•^OH radicals, which are the principal oxidative species responsible for dye degradation, as observed experimentally in this work, altogether with other studies [70]. Secondary mechanisms involve a surface interaction effect: (i) IPA competes with dissolved oxygen for the adsorption sites, potentially reducing the formation of superoxide radical anions (O_2_^•−^) and ultimately the ^•^OH radicals [69,70]; (ii) at the same time, when occupying the ZnO surface, IPA molecules also partially prevent dye molecules from adsorption and subsequent degradation by reactive species generated on the ZnO surface [71]. A residual degradation of 5.4–9.5% (Figure 8b) persisted in the presence of IPA, suggesting the existence of secondary degradation pathways, likely involving direct hole oxidation or alternative reactive radical species [72]. The proposed mechanism, based on our own experimental observations, together with the commonly accepted literature data [72], involves the following:

(1) Photoexcitation and charge carrier generation, in the conduction band and valence band, respectively:
  ZnO + hν → e^−^(CB) + h⁺ (VB) (2)
(2) Formation of reactive oxygen species (ROS):
  h⁺ + H_2_O → ^•^OH + H⁺ (3)
  e^−^ + O_2_ → O_2_^•−^ (4)
  2O_2_^•−^ + 2H⁺ → H_2_O_2_ + O_2_ (5)
  H_2_O_2_ + e^−^ → ^•^OH + OH^−^ (6)
(3) Dye degradation pathways:
  - Primary pathway (>90%): (^•^OH-mediated);
  - Secondary pathways (<10%) (O_2_^•−^, h⁺, and H_2_O_2_ mediated processes).

#### 2.3.4. Kinetics’ Analysis: Applicability of the Langmuir–Hinshelwood Model

The Langmuir–Hinshelwood (L-H) model was applied to elucidate the kinetics of methylene blue (MB) degradation using ZnO nanoparticles as photocatalyst. This model is essential for elucidating reaction mechanisms, optimizing photocatalytic processes, and providing insights into both adsorption and reaction dynamics. Indeed, multiple studies have corroborated the usefulness of this model. According to Villarreal et al. [73], MB degradation using ZnO NPs synthesized from Peumus boldus extract closely adheres to the L-H model, emphasizing the concurrent roles of adsorption and surface reaction kinetics. The versatility of the Langmuir–Hinshelwood (L-H) kinetic model extends to various ZnO-based photocatalytic systems. Nisar et al. [74] demonstrated the effectiveness of the model for ZnO-reduced graphene oxide (rGO) composites, conclusively showing that the L-H model outperformed the Eley–Rideal mechanism in accurately characterizing reaction kinetics. Similarly, Wang et al. [75] validated the L-H kinetic approach for rectorite-based magnetic ZnO composites during methylene blue (MB) degradation, while Elsayed et al. [76] further corroborated these findings by confirming that ZnO/nanohydroxyapatite composites precisely followed the L-H first-order kinetic model during MB photocatalytic elimination. These experimental observations underscore the adaptability of the model across various systems, highlighting its significance in photocatalytic research. In this study, the (L-H) kinetic model was employed to elucidate the photocatalytic degradation of MB using the synthesized ZnO NPs. The degradation rate (r) was correlated with substrate concentration (C) and reaction time (t) using the L-H Equation (7):

(7) $$r=-\frac{dC}{dt}=\frac{k_{r}K_{ad}C}{1+K_{ad}C}$$

where k_r_ is the reaction rate constant, and K_ad_ is the adsorption equilibrium constant [77].

Under low surface coverage and dilute reactant conditions, this simplifies to a pseudo-first-order model Equation (8):

(8) $$Ln\frac{C0}{C}=K_{app}\;\times \;t$$

**Figure 9:**
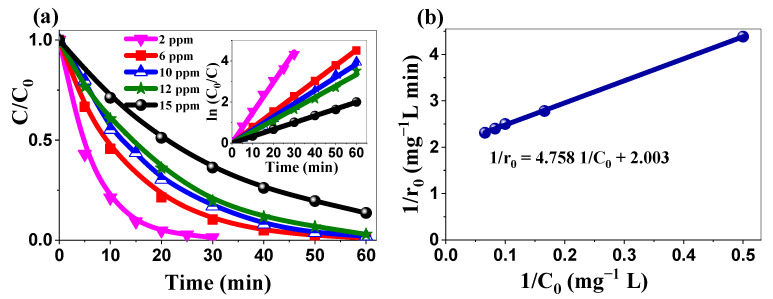
(**a**) Photocatalytic degradation kinetics of MB at various initial concentrations (inset: linear variation of ln(C₀/C) vs. time for different initial concentrations) and (**b**) Langmuir–Hinshelwood kinetics plot for MB degradation ([ZnO NPs] = 1 g/L, pH 7.36).

Photocatalysis of MB was conducted using biosynthesized ZnO NPs (1 g/L) at an initial natural pH of 7.36 under UV irradiation. Figure 9a shows a linear relationship between ln(C_0_/C) and the irradiation time for various initial MB concentrations (2–15 mg/L), with high linear determination coefficients (R^2^ ≥ 0.997), confirming pseudo-first-order kinetics (Table 4).

To validate the L-H model over a wider range of surface coverage, the linear form of the original relation was employed in Equation (9):

(9) $$\frac{1}{r_{0}}=\frac{1}{k_{r}K_{ad}}\frac{1}{C_{0}}+\frac{1}{k_{r}}$$

The initial degradation rate (1/r_0_) was determined by measuring the initial slope (slope at the origin) of the concentration–time curve for each of the five assays, which differed by their initial MB concentration. The plot of 1/r_0_ versus 1/C_0_ (Figure 9b) shows a nearly perfect linear fit, with R^2^ = 0.999, confirming the suitability of the Langmuir–Hinshelwood (L-H) model. The kinetic parameters were determined as K_ad_ = 0.421 mg^−1^ L and k_r_ = 0.5 mg L^−1^ min^−1^. A high K_ad_ value suggests strong MB adsorption onto the ZnO NP surface; whereas, the high k_r_ value indicates a rapid surface reaction, resulting in an overall high photocatalytic efficiency. This comprehensive analysis confirms once more the status of the L-H model as a robust framework for elucidating organic compound degradation kinetics. The combination of high adsorption capacity and fast surface reaction explains the significant photocatalytic activity of the biosynthesized ZnO NPs and provides valuable insights for optimizing photocatalytic processes. These findings highlight the potential of our biosynthesized ZnO NPs for practical wastewater treatment applications.

#### 2.3.5. Recyclability Performance Evaluation

**Figure 10:**
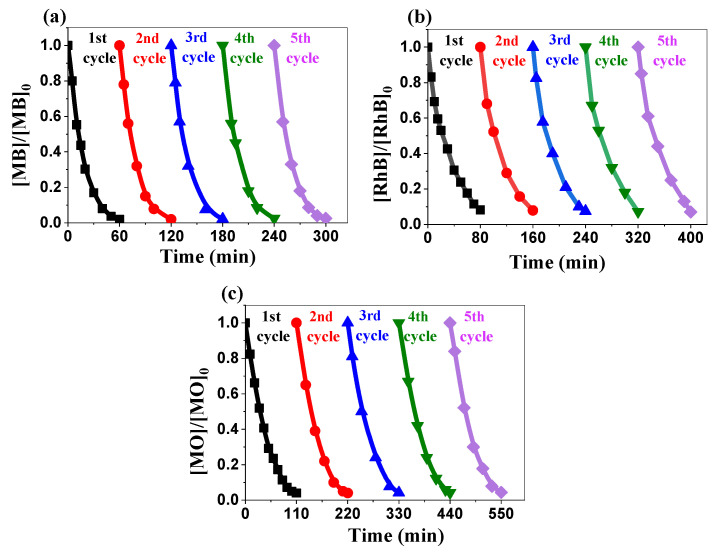
Recyclability of the biosynthesized ZnO NPs: (**a**) MB, (**b**) RhB, and (**c**) MO.

Figure 10a–c shows the excellent recyclability of the synthesized ZnO NPs over five consecutive cycles. The degradation efficiencies remained consistently high, above 97% for methylene blue, 93% for rhodamine B, and 95% for methyl orange. This remarkable stability over multiple cycles is crucial for practical applications, as it significantly reduces the operational costs and environmental impacts associated with catalyst replacement. The maintained high efficiency suggests the minimal loss of active sites or structural degradation during the photocatalytic process. This consistent performance across multiple cycles highlights the stability of biosynthesized ZnO. Together with its efficiency, it stresses its economic viability and potential for applications in wastewater treatment.

### 2.4. DPPH Radical Scavenging Activity of Biosynthesized ZnO Nanoparticles

The development of effective antioxidant materials is crucial for various biomedical applications, particularly in the context of the antiradical properties of biosynthesized ZnO oxide nanoparticles under UV radiation. These nanoparticles demonstrated a significant capacity to neutralize 2,2-diphenyl-1-picrylhydrazyl (DPPH) radicals, rendering them promising candidates for pharmaceutical, cosmetic, and functional food applications. The antioxidant mechanism of ZnO NPs operates through a sophisticated interplay between photocatalytic and chemical processes. Under UV irradiation, ZnO NPs generate electron–hole pairs, leading to the formation of reactive oxygen species (ROS), including superoxide (O_2_^•−^) and hydroxyl (^•^OH) radicals [78]. These photogenerated species, in conjunction with direct electron transfer from the ZnO conduction band, facilitate the reduction in DPPH radicals to their nonradical form, demonstrating a dual-mechanism approach to radical neutralization [79,80]. Several factors influence the antioxidant efficacy of ZnO NPs. UV exposure enhances antiradical properties by increasing ROS generation, as demonstrated by Pairoj et al. [81], who also reported optimal antioxidant activity at 200 μg/mL. Particle size plays a crucial role in antiradical activity, with smaller nanoparticles providing larger surface areas for DPPH radical scavenging [82]. The antioxidant efficacy of the biosynthesized ZnO NPs synthesized in this study was quantitatively evaluated through EC_50_ measurements, defined as the concentration required for a 50% reduction in DPPH^•^ concentration. This parameter serves as a standardized metric for expressing the antioxidant capacity, and the results are presented in Table 5.

**Figure 11:**
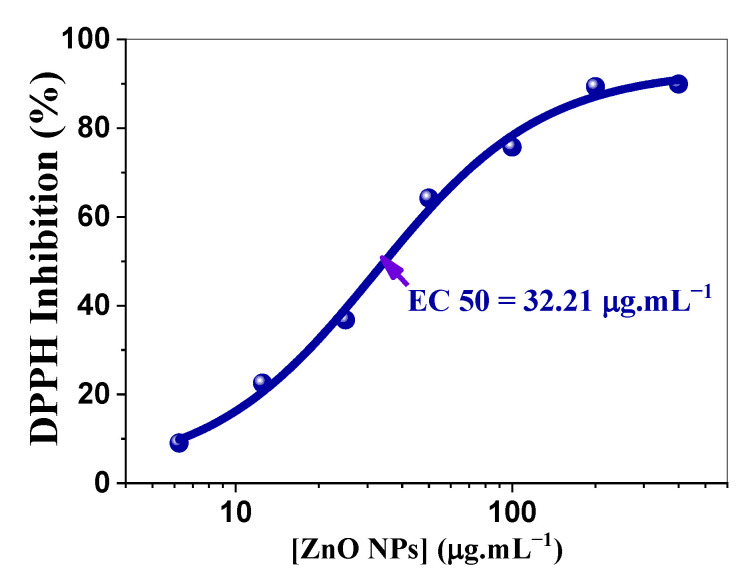
DPPH scavenging activity plot.

The data revealed a clear concentration-dependent response, commencing with the minimal inhibition of (9.05 ± 0.29)% at 6.25 μg/mL, followed by a marked increase in activity within the 12.5–50 μg/mL range, indicating an optimal concentration window. The inhibition reached (75.72 ± 0.38)% at 100 μg/mL and plateaued at approximately 89% at 200 μg/mL. The increase in activity with the increasing concentration of ZnO NPs can be attributed to the increase in the number of active sites. This phenomenon resulted in two concurrent effects: an increase in the quantity of adsorbed DPPH molecules and an increase in the number of photogenerated electrons. These effects synergistically enhanced the inhibitory activity of NPs, resulting in a direct increase in their overall antioxidant performance. At concentrations exceeding 200 mg/L, the antioxidant activity was stabilized, indicating no further improvements. This stagnation may be attributed to the UV-blocking properties of ZnO NPs at elevated concentrations. The outer layers of the nanoparticles create a screening effect that reduces UV penetration into the inner layers, thereby limiting the capacity of the system to effectively scavenge further DPPH radicals. Consequently, the rate of photogenerated electrons remained constant, which could explain the observed stagnation at 89% antioxidant activity for NPs beyond 200 mg/L. Furthermore, the competing effect of DPPH and dissolved oxygen for the active sites may be the root cause that prevents the complete inhibition of the DPPH radical, thus limiting the inhibition efficiency of ZnO NPs at this maximum level of 89%. Figure 11 illustrates the dose-dependent DPPH radical scavenging activity of ZnO NPs, displaying a sigmoidal curve with an EC_50_ value of 32.21 µg.mL^−^¹.

A comparative analysis of various ZnO NPs’ preparations (Table 6) revealed significant variations in antioxidant efficacy. Our biosynthesized ZnO NPs demonstrated superior antioxidant activity with an EC₅₀ value of (32.21 ± 4.62) μg/mL, substantially outperforming precipitation method ZnO NPs (EC₅₀ = 97.65 μg/mL) [82] and exhibiting comparable efficiency to commercial ZnO NPs (EC₅₀ = 36.6 μg/mL) [83]. Notably, ZnO NPs prepared using the sol-gel method and calcined at 300 °C exhibited the highest antioxidant activity, with an EC₅₀ value of 7.8 μg/mL [83].

The superior antioxidant performance of the starch-mediated sol-gel-synthesized ZnO NPs may be due to their optimized particle size distribution, enhanced surface area, increased density of active radical scavenging sites, or improved photocatalytic response to UV radiation. These findings highlight the significant potential of biosynthesized ZnO NPs for pharmaceutical and cosmetic applications where oxidative stress mitigation is crucial.

### 2.5. Electrochemical Study

#### 2.5.1. Cyclic Voltammetry

**Figure 12:**
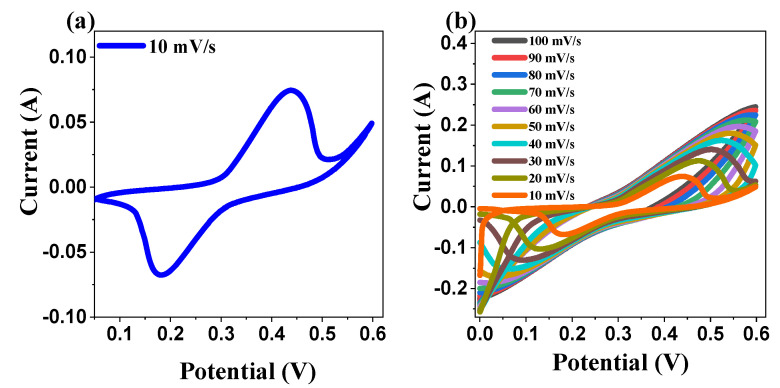
Electrochemical characterization of zinc oxide electrode in a three-electrode system (**a**) CV curve at a scan rate of 10 mV/s and (**b**) CV curves of the ZnO NPs electrode at various scan rates.

To investigate the electrochemical behavior of our ZnO electrode, we performed a series of cyclic voltammetry (CV) experiments at various scan rates, as presented in Figure 12a,b. These experiments aimed to analyze the charge storage and transfer properties of the material under different conditions. The presence of well-defined redox peaks in the cyclic voltammetry (CV) curves provides strong evidence for the pseudocapacitive nature of the ZnO electrode and demonstrates its capacity for rapid, reversible redox reactions at the surface, which is a crucial characteristic for energy storage applications. A significant shift in the peak positions was observed when the scan rate was increased from 10 mV/s to 100 mV/s. In particular, the anodic peaks shifted towards higher potentials, while the cathodic peaks migrated towards lower potentials (Figure 12b). This shift is a clear indication of the increase in the internal resistance of the system at higher scan rates. This phenomenon is commonly observed in electrochemical systems, because higher scan rates do not allow sufficient time for ion diffusion and charge transfer at the electrode-electrolyte interface, leading to delays and peak shifts in the electrochemical processes. These results are consistent with those of previous studies in the field that observed similar behaviors in various electrode materials under fast scan conditions [84,85]. Furthermore, the observed increase in the current response with increasing scan rate suggests that the kinetics of the redox reactions increase as the scan rate increases. This increase in the current response with higher scan rates is crucial for the application of the material in supercapacitors, as it demonstrates that the ZnO electrode can accommodate higher charge/discharge rates without significant deterioration in performance. The faster kinetics of redox reactions at the electrode surface mean that the material can provide high-power output in practical applications, a vital feature for supercapacitors used in energy storage and fast charge–discharge cycling. Additionally, this behavior may indicate an increased number of active sites accessible for redox reactions, suggesting that ZnO may be an effective candidate for high-performance electrochemical energy storage devices. Overall, the results obtained from the cyclic voltammetry experiments provide valuable insights into the electrochemical performance of the ZnO electrode and demonstrate its promising potential for use in supercapacitors and other energy storage technologies.

**Figure 13:**
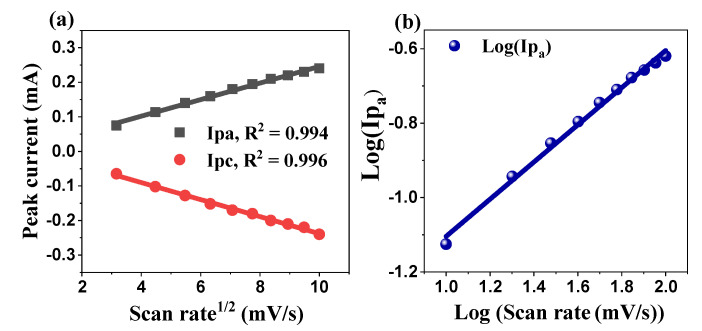
(**a**) Plot of peak current versus the square root of the scan rate for the ZnO NPs electrode and (**b**) electrochemical kinetic.

Further analysis of the cyclic voltammetry (CV) data revealed a strong linear correlation between the peak currents and the square root of the scan rate, as shown in Figure 13a. This relationship was confirmed by high R^2^ values of 0.994 and 0.996 for the anodic and cathodic processes, respectively, providing substantial evidence that the electrochemical reaction was predominantly diffusion-controlled. This linearity suggests that the charge–transfer process is governed by the diffusion of ions to the electrode surface, a characteristic feature of materials exhibiting diffusion-controlled behavior, such as battery-type electrodes. To gain a deeper understanding of the charge storage mechanism, we further investigated the relationship between the logarithm of the anodic peak current (log Ip) and the logarithm of the scan rate (logV) (Figure 13b). This relationship can be described by the power–law Equation (10):

Ip_a_ = a × v^b^ (10)

where (a) is a constant, and (b) is the slope. The value of the slope (b) provides valuable insights into the dominant charge storage mechanism in the system: when b ≈ 1, it indicates a surface-controlled process that is characteristic of electric double-layer capacitance (EDLC) behavior. In this regime, ion adsorption/desorption at the electrode–electrolyte interface dominates, and charge storage is primarily capacitive. This is typical for supercapacitors, in which charge is stored through electrostatic interactions at the surface of the electrode. When b ≈ 0.5, the slope is close to 0.5, suggesting a diffusion-controlled process, which is typical of battery-type behavior. In this case, the charge storage mechanism is dominated by redox reactions at the electrode surface, with the rate of ionic diffusion within the electrode material being the limiting factor. This is often observed in materials, such as transition metal oxides and sulfides, where charge storage is dependent on the diffusion of ions into the bulk of the material during the redox reaction. The deviation of (b) from these ideal values: If the value of (b) deviates from 1 or 0.5, it may suggest that both capacitive and diffusion-controlled processes contribute to the overall charge storage behavior. This mixed mechanism is often observed in materials that exhibit both electric double-layer capacitance and pseudo-capacitance (due to Faradaic redox reactions), such as certain metal oxide electrodes. The power–law relationship and slope (b) provide significant information regarding the nature of the electrochemical process occurring at the electrode surface [86]. The ability to distinguish between surface-controlled and diffusion-controlled processes is crucial for optimizing materials for energy storage applications. For instance, surface-controlled processes are ideal for supercapacitors because of their fast charge/discharge rates; whereas, diffusion-controlled processes are typically more relevant for batteries where higher energy densities are often sought [87]. These insights can help in understanding the fundamental behavior of materials such as ZnO in electrochemical energy storage devices and in optimizing their performance for practical applications, such as supercapacitors and hybrid energy storage systems.

#### 2.5.2. Galvanostatic Charge–Discharge Studies

**Figure 14:**
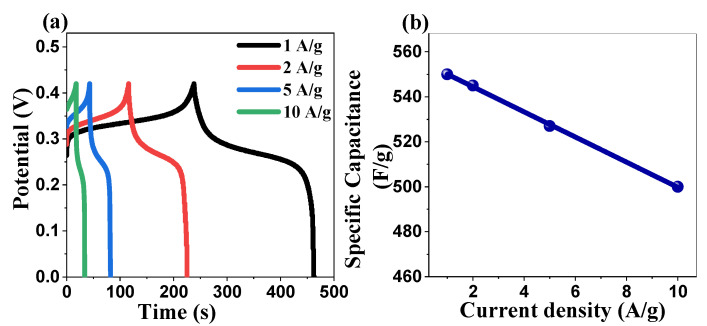
(**a**) GCD curves at various current densities for the ZnO NPs electrode and (**b**) specific capacitance at different current densities.

To assess the charge storage capacity and rate capability of the ZnO electrode, galvanostatic charge–discharge (GCD) experiments were conducted (Figure 14a). The GCD curves exhibited a characteristic nonlinear triangular profile, further corroborating the pseudocapacitive behavior of the ZnO electrode [88,89,90]. Notably, a maximum specific capacitance of 550.372 F/g was achieved at a current density of 1 A/g, with an energy density of 12.77 W h/kg at 99.32 W/kg of power density, demonstrating its competitiveness with other metal oxide-based supercapacitors reported in the literature. An inverse relationship between capacitance and current density was observed (Figure 14b), likely attributable to limited electrolyte ion accessibility within the electrode structure at higher charge–discharge rates [91]. This behavior suggests the potential for further optimization of the electrode architecture to enhance high-rate performance.

#### 2.5.3. Electrochemical Impedance Spectroscopy Investigation

**Figure 15:**
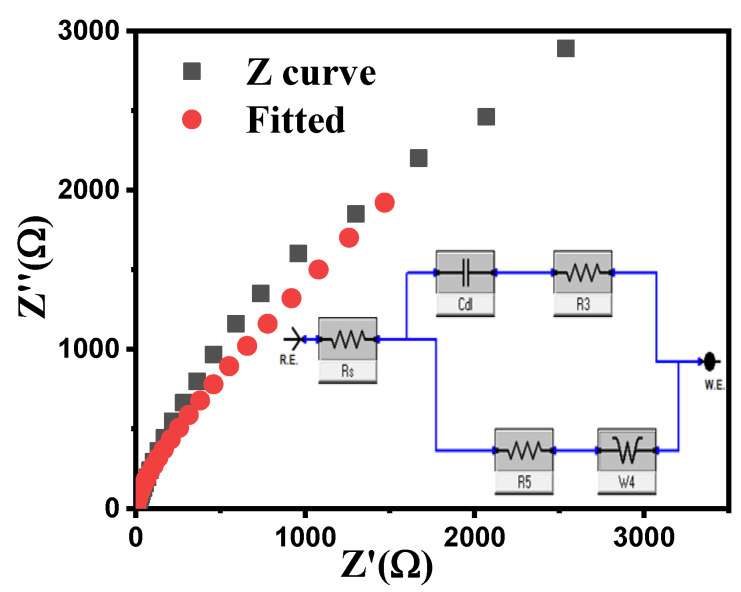
Electrochemical impedance spectroscopy analysis of ZnO NPs electrode material.

Electrochemical impedance spectroscopy (EIS) analysis was conducted to elucidate the electrode–electrolyte interface dynamics and charge–transfer processes. The Nyquist plot (Figure 15) exhibits two distinct regions: a small semicircle in the high-frequency domain and a near-vertical line in the low-frequency domain. The semicircle corresponds to the charge transfer resistance at the electrode–electrolyte interface, while the vertical line indicates ideal capacitive behavior [92,93]. The small semicircle diameter suggests low charge–transfer resistance, which is advantageous for rapid charge/discharge processes. Furthermore, the slight deviation from perfect verticality indicated the presence of a finite diffusion resistance within the electrode structure.

#### 2.5.4. Comparative Analysis and Discussion

This comprehensive electrochemical investigation demonstrated the significant potential of ZnO nanoparticles (NPs) as electrode materials for next-generation supercapacitors. The ZnO NPs exhibited a remarkable specific capacitance of 550 F/g at a current density of 1 A/g in a 2 M KOH electrolyte. This high specific capacitance value is particularly noteworthy, because it highlights the ability of ZnO NPs to store a significant amount of charge per unit mass, which is crucial for supercapacitor performance. Additionally, the material displayed a characteristic pseudocapacitive behavior, which means that the charge storage mechanism is partially due to fast, reversible redox reactions on the electrode surface. This behavior is typically observed in materials that undergo Faradaic reactions in addition to the electrostatic charge storage observed in electric double-layer capacitors (EDLCs). To further contextualize these findings and assess the performance of ZnO NPs relative to other state-of-the-art materials in the field of supercapacitors, we conducted a systematic comparison with recent literature reports [94,95,96,97]. The comparison, summarized in Table 7, highlights the diverse approaches used to synthesize ZnO-based electrodes and their corresponding electrochemical performance. The specific capacitance values from various studies are presented, providing insights into how the current study’s findings stand in relation to others.

The comparison of the specific capacitance values reveals that the ZnO NPs synthesized in this study exhibit a competitive capacitance of 550 F/g, positioning them favorably among other ZnO-based supercapacitors in the recent literature. While some studies reported higher capacitances, such as 708.75 F/g achieved by hybrid machining, the current study’s results are still highly promising, especially considering the synthesis method used (starch-mediated sol-gel), which is simple, cost-effective, and scalable for practical applications. Additionally, the use of a 2 M KOH electrolyte enhanced the performance by providing higher ionic conductivity, allowing for better charge transport and electrochemical performance. This comparison underscores the importance of both synthesis methods and electrolytes in determining the performance of ZnO-based supercapacitors and highlights the potential of ZnO NPs for use in next-generation energy storage devices.

### 2.6. Cytotoxicity Assessment of ZnO NPs

Cytotoxicity analysis revealed an LC_50_ value of 1648 μg/mL (95% confidence interval: 1127.548–3472.362 μg/mL), indicating low acute toxicity according to the classification by Meyer et al. [98], wherein LC_50_ values exceeding 1000 μg/mL are considered nontoxic. A distinct dose-dependent relationship between the ZnO NPs concentration and nauplii mortality was observed, with mortality rates increasing from 6.67 ± 5.77% at 125 μg/mL to 53.33 ± 5.77% at 2000 μg/mL (Table 8).

**Figure 16:**
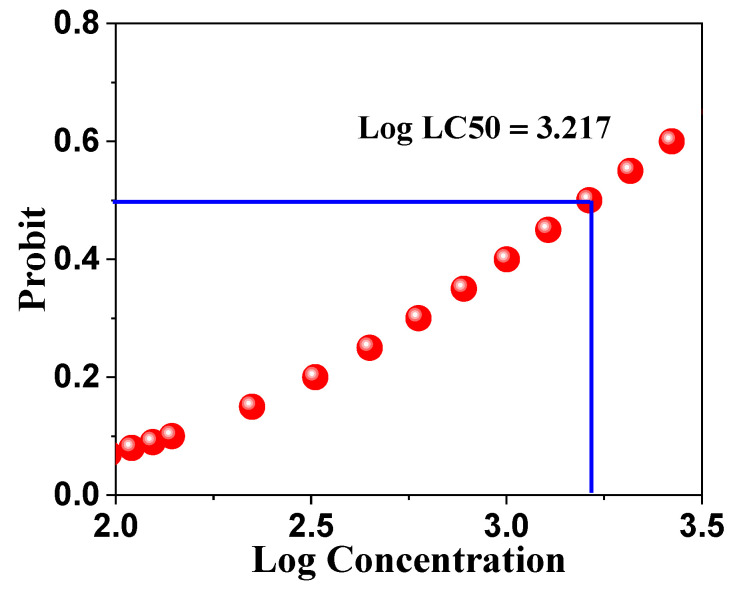
Probit analysis of Artemia salina mortality versus log concentration of ZnO NPs for LC50 determination.

The probit plot demonstrated a strong linear relationship between log concentration and probit of mortality (R^2^ = 0.981), underscoring the reliability of the LC_50_ determination (Figure 16). This high R^2^ value indicates the robustness of the experimental design and validity of the toxicity assessment, providing a solid foundation for subsequent risk assessment studies.

The reduced toxicity profile of these starch-mediated sol-gel synthesized ZnO NPs points toward a potential for safe applications in various fields, thereby presenting new opportunities for ZnO NPs with diminished environmental and biological impacts.

## 3. Methods and Materials

ZnO NPs were synthesized utilizing zinc nitrate hexahydrate (Zn(NO_3_)_2_·6H_2_O, 98% purity, Sigma-Aldrich, St. Louis, MO, USA) as the zinc precursor. Soluble starch (Formula: (C_6_H_10_O_5_)_n_, CAS: 9005-84-9) was obtained from Merck (Darmstadt, Germany) as the stabilizing agent. Distilled water served as the reaction medium. For comparative photocatalytic degradation studies, commercial ZnO nanopowder (Sigma-Aldrich) was employed as a benchmark; whereas, methylene blue, methyl orange, and rhodamine B (all from Sigma-Aldrich) were used as model dye pollutants. Isopropanol (Sigma-Aldrich) was employed as a scavenger in the photocatalytic experiments. For electrochemical studies, acetylene black, polyvinylidene fluoride (PVDF, 99% purity), and N-methyl-2-pyrrolidone (NMP, 99.5% purity) were acquired from Sigma-Aldrich. KOH (85% purity) and HCl (37% purity) were purchased from Merck. Antioxidant activity assays were conducted using 2,2-diphenyl-1-picrylhydrazyl (DPPH) and methanol (both from Sigma-Aldrich). For the toxicity assessment, brine shrimp (Artemia salina) cysts were obtained from a local commercial supplier in Algeria. All chemicals were used as received without further purification.

### 3.1. Zinc Oxide Nanoparticle Synthesis

**Figure 17:**
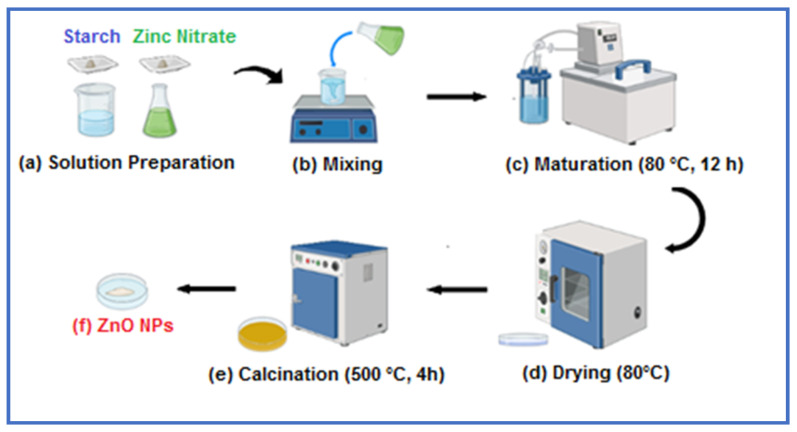
Schematic representation of ZnO NP synthesis using the sol-gel technique.

A modified sol-gel method, inspired by the methodology reported in [27], was developed and employed for the synthesis of the ZnO nanoparticles. Initially, the starch solution was prepared by dissolving 3.33 g of soluble starch in 50 mL of distilled water under continuous agitation. Concurrently, a 1.5 M aqueous solution of zinc nitrate hexahydrate was prepared. The zinc precursor solution was introduced into the starch solution at a volumetric ratio of 1:3, followed by thorough mixing for 30 min at ambient temperature. The resulting mixture was transferred to a temperature-controlled reactor vessel maintained at 80 °C and subjected to constant agitation for 12 h. Subsequently, the gel-like solution was transferred to a glass petri dish and dried in a precision oven at 80 °C for 12 h. Finally, the dried gel was calcined in a muffle furnace at 500 °C for 4 h (Figure 17).

### 3.2. Point of Zero Charge Measurement of ZnO Nanoparticles

The point of zero charge (pH_PZC_) of the bio-synthesized ZnO NPs was determined using the pH drift method adapted from [99] with minor modifications. A series of experiments were conducted in which ZnO samples (50 mg) were systematically dispersed in 20 mL of 0.050 mol/L NaCl solution in separate 50 mL falcon tubes. The initial pH (pHi) of each suspension was precisely adjusted within a range of 3–12 using standardized 0.10 mol/L solutions of HCl and NaOH. To ensure experimental integrity, the tubes were hermetically sealed, immediately after pH adjustment. The suspensions were then subjected to continuous agitation in a temperature-controlled shaker maintained at 298 K for an equilibration period of 48 h. Subsequently, the samples were centrifuged at 7000 rpm for 15 min to achieve phase separation. The pH measurements were recorded before (pH_i_) and after (pH_f_) the equilibration period using a HI2002-01 edge^®^ digital pH meter (HANNA Instruments, Woonsocket, RI, USA). pH_PZC_ was identified at the intersection point between the ΔpH (pH_f_-pH_i_) versus pH_i_ plot and the zero line, representing the pH at which the net surface charge is neutral.

### 3.3. Characterization Methods

The physicochemical properties of the synthesized ZnO NPs were comprehensively characterized using advanced analytical techniques. The optical properties of the samples were investigated by UV-visible spectroscopy using a UV-Visible spectrophotometer (Shimadzu Corporation, Kyoto, Japan). Field emission scanning electron microscopy (FE-SEM, Gemini 550, Zeiss, Oberkochen, Germany) was used to analyze the morphology and conduct elemental mapping of the synthesized ZnO NPs. The same apparatus was employed for scanning transmission electron microscopy (STEM) to provide additional high-resolution structural characterization. The crystalline structure and phase purity were analyzed by X-ray diffraction (XRD) using a D8 ADVANCE diffractometer (Bruker, Billerica, MA, USA) equipped with Cu-Kα radiation (λ = 1.5406 Å). The chemical bonding characteristics were investigated using Fourier-transform infrared (FT-IR) spectroscopy performed on a Spotlight 400 FTIR Imaging System (PerkinElmer, Waltham, MA, USA). The textural properties of the ZnO NPs were analyzed using a Gemini VII Surface Area and Porosity Analyzer (Micromeritics, Mönchengladbach, Germany) via N_2_ adsorption–desorption measurements at 77 K. The Brunauer–Emmett–Teller (BET) method was used to calculate the specific surface area, and the total pore volume was determined from the adsorption data. The average pore width was calculated using the Barrett–Joyner–Halenda (BJH) method.

### 3.4. Photocatalytic Degradation

Photocatalytic activity was evaluated using a custom-designed photoreactor. The apparatus comprised a cylindrical quartz vessel (150 mL capacity) equipped with a 15-watt low-pressure mercury UV lamp (Philips, Amsterdam, The Netherlands, λmax = 365 nm). Three model organic pollutants were selected: cationic methylene blue (MB), rhodamine B (RhB), and anionic methyl orange (MO) (Table 9). For each experiment, 100 mL of the dye solution (10 mg/L) was combined with 0.1 g of a ZnO photocatalyst (NPs synthesized in this study or commercial powder). The suspension was magnetically stirred in the dark for 45 min to establish an adsorption–desorption equilibrium prior to irradiation. Aliquots (3 mL) were extracted at regular time intervals and centrifuged at 7000 rpm for 10 min to separate the catalyst. The dye degradation was monitored using UV-Vis spectroscopy by measuring the absorbance at the maximum absorption wavelength of MB (652 nm), RhB (553 nm), and MO (462 nm). The concentration reached after equilibration was considered as the initial concentration in these essays (C_0_) and served as a reference for subsequent measurement. C_0_ and concentration at time t were determined spectrophotometrically. The dark adsorption capacity of methylene blue (MB) was 8% at pH 7.3 and 12% at pH 11, with comparable values observed for rhodamine B (RhB) and methyl orange (MO). Experiments were initially conducted at neutral pH without adjustment to facilitate the direct comparison of the photocatalytic efficiencies of the various dyes. For experiments conducted at pH 11, the dye solution was adjusted utilizing a 0.1 M NaOH solution prior to the addition of ZnO nanoparticles, followed by dark equilibration (45 min) and then UV light exposure for photocatalysis evaluation. All measurements were performed in triplicate. The degradation percentage was calculated using the following Equation (11):

(11) $$\mathrm{Degradation}\;(\%)=\frac{\left(C_{0}-C_{t}\right)}{C_{0}}\times 100$$

where C_0_ is the initial concentration after dark adsorption, and C_t_ is the concentration at time t.

To elucidate the role of hydroxyl radicals (•OH) in the photocatalytic degradation mechanism, additional experiments were conducted utilizing isopropanol (IPA) as a radical scavenger. The photocatalytic degradation experiments were replicated under identical conditions as for previous essays, except for the addition of 0.5% *v*/*v* IPA to the reaction mixture containing MB, RhB, or MO dyes (10 ppm) and ZnO (1 g/L). The degradation efficiencies and kinetic parameters were subsequently compared with those obtained without IPA to assess the contribution of hydroxyl radicals to the photocatalytic process.

### 3.5. Recyclability Testing Procedure

The long-term stability and recyclability of the ZnO photocatalyst were evaluated through consecutive dye degradation essays. A 10 ppm dye solution with 1 g/L of ZnO catalyst was prepared and subjected to UV irradiation until complete degradation occurred. After each cycle, the ZnO catalyst was recovered via filtration, washed, dried, and subsequently reused in a fresh dye solution. This procedure was repeated for five consecutive cycles. The degradation kinetics were monitored by observing the C/C_0_ ratio as a function of the irradiation time. The experimental conditions were kept constant throughout the study to ensure that any observed performance variation was solely attributable to catalyst recycling.

### 3.6. DPPH Radical Scavenging Assay and EC_50_ Determination

The antiradical activity of the ZnO nanoparticles was evaluated using a modified DPPH radical scavenging assay [100]. ZnO nanoparticle suspensions were prepared at concentrations ranging from 6.25 to 400 μg/mL. DPPH solution (0.06 mg/mL in methanol) was prepared and stored at −20 °C in the absence of light. The absorbance of the DPPH solution was adjusted to 0.5 at 517 nm. For the assay, 2 mL of DPPH solution was combined with 0.5 mL of each ZnO nanoparticle suspension. The mixtures were subjected to UV irradiation (λ = 365 nm) for 2 min, followed by centrifugation at 7000 rpm for 5 min. The absorbance of the supernatant was measured at a wavelength of 517 nm. The experiments were limited to 2 min of reaction because of the high scavenging rate, established in preliminary essays. The percentage inhibition was calculated using the following Equation (12):

(12) $$\mathrm{Inhibition}\;(\%)=\frac{\left(A_{control}-A_{sample}\right)}{A_{control}}\times 100$$

where A_control_ represents the absorbance of the blank DPPH solution, and A_sample_ represents the absorbance of the DPPH solution after its exposition to UV irradiation in the presence of ZnO NPs.

### 3.7. Electrode Preparation

The ZnO electrodes were fabricated using Ni foam (1 × 1 cm^2^) as the current collector. The foam was pretreated by immersion in 1 M HCl and sonication for 30 min, followed by desiccation at 80 °C. A slurry comprising ZnO (85% *w*/*w*), acetylene black (10% *w*/*w*), and PVDF (5% *w*/*w*) in NMP was prepared and applied onto Ni foam. The coated foam was dried at 80 °C to facilitate the removal of NMP. The final mass of the active material coating was 5 mg.

### 3.8. Electrochemical Characterization

Electrochemical measurements were conducted using a three-electrode system in 2 M KOH electrolyte at 25 °C. The ZnO-coated nickel foam functioned as the working electrode, with platinum foil (1 cm × 1 cm) serving as the counter electrode and saturated Ag/AgCl (3 M KCl) as the reference electrode. All the measurements were performed using a GAMRY Reference 3000 potentiostat. Cyclic voltammetry (CV) was conducted at scan rates of 10–100 mV/s over a potential range of 0–0.6 V. Galvanostatic charge–discharge (GCD) measurements were performed at current densities ranging from 1 to 10 A/g. Electrochemical impedance spectroscopy (EIS) was carried out in the frequency range of 10 kHz to 0.01 Hz with a 10 mV amplitude. The specific capacitance (C) was calculated from the GCD curves using Equation (13).

(13) $$C=\frac{\left(I\times \Delta t\right)}{\left(m\times \Delta V\right)}\times 100$$

where I is the discharge current (A), Δt is the discharge time (s), m is the mass of active material (g), and ΔV is the potential window (V). The energy (E, Wh/kg) and power densities (P, W/kg) of the supercapacitor were calculated using Equations (14) and (15).

(14) $$E=\frac{C\times {∆V}^{2}}{2\times 3.6}$$

(15) $$P=\frac{3600\times E}{∆t}$$

### 3.9. Brine Shrimp Lethality Assay (BSLA)

The in vitro cytotoxicity assay was conducted in accordance with the protocol described by Supraja et al. [101] with some modifications. Artemia salina cysts (1 g) were incubated in natural seawater (1 L) obtained from the Mediterranean coast of Algeria. The incubation was maintained at 28 ± 1 °C under constant illumination and aeration for 48 h. Nauplii were separated from the empty shells by filtration to ensure the utilization of viable organisms. Toxicity assessments were performed in triplicates using sterile borosilicate glass test tubes. Each tube contained 2 mL of natural seawater, 2 mL of ZnO NPs suspension, and 10 nauplii. ZnO NPs were evaluated at concentrations of 2000, 1000, 500, 250, 125, and 62.5 μg/mL. A negative control comprising 2 mL of seawater and 2 mL of distilled water was used to assess baseline mortality in the absence of ZnO NPs. Following 24 h exposure at 28 ± 1 °C, nauplii viability was evaluated, with nonviable larvae defined as those immotile for more than 10 s upon gentle stimulation. The median lethal concentration (LC_50_) was determined by Finney’s probit analysis with a 95% confidence interval using SPSS software (version 26). According to Meyer et al. [98], LC_50_ values below 1000 μg/mL are considered toxic; whereas, LC_50_ values above 1000 μg/mL are nontoxic. The percentage mortality (M%) was calculated using the following Equation (16):

(16) $$M\%=[\frac{\left(N₀-N₁\right)}{N₀}\times 100]$$

where N_0_ is the initial number of viable nauplii, and N_1_ is the number of surviving nauplii after exposure.

## 4. Conclusions

This study demonstrates the development of multifunctional ZnO NPs through a starch-based sol-gel synthesis approach that addresses key challenges in nanomaterial production. The starch-mediated synthesis enables an environmentally conscious pathway, while yielding ZnO NPs with distinct characteristics. The synthesized nanoparticles exhibited efficient performance in multiple applications. The crystalline mesoporous structure contributed to the substantial photocatalytic activity, achieving methylene blue degradation in 15 min at pH 11. This efficiency, along with the degradation of various organic dyes following pseudo-first-order kinetics, indicates their potential for environmental remediation applications. For energy storage, the nanoparticles demonstrated notable specific capacitance (550 F/g at 1 A/g) for supercapacitor applications. The UV-induced antiradical properties (EC_50_: 32 μg/mL in DPPH tests) combined with low cytotoxicity (LC_50_: 1648 μg/mL) contribute to the development of biocompatible nanomaterials. Comprehensive characterization and performance evaluation established that starch-mediated synthesis offers a sustainable approach for producing multifunctional ZnO nanoparticles. The integration of photocatalytic, supercapacitive, and UV-induced antiradical properties within environmentally conscious nanomaterial systems significantly contributes to sustainable nanotechnology. These findings provide a foundation for developing multifunctional nanomaterials that can address environmental remediation, energy storage, and biomedical challenges, while maintaining sustainability and biocompatibility standards.

## Figures and Tables

**Table 1:** Calculated structural parameters of ZnO NPs.

2θ (°)	(hkl)	FWHM (°)	a (nm)	c (nm)	D (nm)	D_mean_ (nm)
31.932	(100)	0.37	0.323		22.332	22.2 ± 0.8
34.578	(002)	0.359		0.518	23.176	
36.415	(101)	0.394			21.226	

**Table 2:** Apparent rate constants (k_app_) for commercial and synthesized ZnO NPs.

k_app_ (min^−1^)			
Photocatalyst	MB	RhB	MO
C-ZnO	0.227	0.293	0.115
ZnO NPs	0.064	0.03	0.027

**Table 3:** Comparative analysis of ZnO-based photocatalysts for dyes degradation.

Dye	Catalyst	Synthesis Method	[Dye]_0_ (ppm)	k_app_ (min^−1^)	Deg (%)	Time (min)	[Ref]
MB	ZnO NPs	sol gel	10	0.064 pH 7.3	98	60	Present work
	ZnO NPs	sol gel	10	0.33 pH 11	100	15	Present work
	ZnO	sol-gel	10	0.0106	86	140	[62]
	2% Fe-ZnO	sol-gel	10	0.0107	92	140	[62]
	Cu₂O/ZnO/GO (5%)	coprecipitation and hydrothermal	5	-	91.4	120	[63]
	ZnO	sonication	10	0.009	54	80	[60]
	NiO-ZnO	sonication	10	0.015	72	80	[60]
	7.5% Cu-ZnO	impregnation	20	0.137 (45 °C)	99	15	[64]
RhB	ZnO NPs	sol-gel	10	0.03	92	80	Present work
	ZnO NPs	solution phase	10	0.034	95	70	[65]
	5% Se-ZnO NPs	sol-gel precipitation	10	0.014	98.23	150	[66]
MO	ZnO NPs	sol gel	10	0.027	96	110	Present work
	ZnO NPs	laser-assisted chemical bath	20	0.00417	85	120	[67]
	1% Ce-ZnO NPs	laser-assisted chemical bath	20	0.015	92	120	[67]

**Table 4:** Apparent pseudo-first-order rate constant for MB degradation at different initial concentrations.

**C_0_ (mg/L)**	2	6	10	12	15
**K_app_ (min^−1^)**	0.1481	0.075	0.064	0.055	0.033
**R^2^**	0.998	0.999	0.997	0.997	0.999

**Table 5:** DPPH radical scavenging activity of biosynthesized ZnO NPs.

[ZnO] (µg/mL)	6.25	12.5	25	50	100	200	400	EC50 (µg/mL)
Inhibition (%)	9.05 ± 0.29	22.56 ± 0.38	36.7 ± 0.11	64.24± 0.19	75.72 ± 0.38	89.36 ± 0.11	89.93 ± 0.11	32.21± 4.62

**Table 6:** Comparative EC_50_ values of ZnO NPs.

Study	Material	EC_50_ (μg/mL)	Synthesis Method
Current study	ZnO NPs	32.21	Starch-mediated sol-gel
[82]	ZnO submicron particles	97.65	Precipitation method
[83]	ZnO NPs	7.8	Sol-gel, calcined at 300 °C
[83]	Commercial ZnO	36.6	Commercial (100 nm diameter)

**Table 7:** Comparison of specific capacitance values for ZnO-based supercapacitors.

Study	ZnO Material	Specific Capacitance (F/g)	Current Density (A/g)	Electrolyte	Synthesis Method
Current Study	ZnO NPs	550	1	KOH (2 M)	Starch sol-gel
[94]	Curcuma amada-functionalized ZnO	457	1	KOH (1 M)	Biosynthesis
[95]	ZnO NPs	214	1	Na_2_SO_4_ (1 M)	Hydrothermal
[96]	ZnO NPs	170.6	1	KOH (1 M)	Glycine-based auto-combustion
[97]	ZnO NPs	708.75	1	KOH (1 M)	Hybrid machining

**Table 8:** Effect of zinc oxide nanoparticle ZnO NPs concentration on Artemia salina nauplii mortality after 24 h.

[ZnO] (μg/mL)	Number of Dead Nauplii (After 24 h)			Mortality %	LC_50_ (μg/mL)	95% Confidence Interval
	Replication 1	Replication 2	Replication 3			
0	0	0	0	00.00 ± 00.00	1647.696	[1127.548–3472.362]
125	1	0	1	6.67 ± 5.77		
250	2	1	3	20.00 ± 10.00		
500	3	3	2	26.67 ± 05.77		
1000	4	5	3	40.00 ± 10.00		
2000	5	6	5	53.33 ± 05.77		

**Table 9:** Physicochemical characteristics of organic pollutants investigated in this study.

Compound	Methylene Blue (MB)	Rhodamine (RhB)	Methyl Orange (MO)
Structure	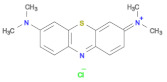	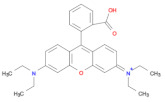	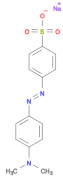
Molecular formula	C16H18N3SCl	C28H31ClN2O3	C14H14N3NaO3S
λmax	652 nm	553 nm	462 nm

## Data Availability

Data are contained within the article and Supplementary Materials.

## Supplementary Materials

The following supporting information can be downloaded at: https://www.mdpi.com/article/10.3390/ijms26020859/s1.
