# Supplementary figures and images for: Starch-Assisted Eco-Friendly Synthesis of ZnO Nanoparticles: Enhanced Photocatalytic, Supercapacitive, and UV-Driven Antioxidant Properties with Low Cytotoxic Effects
Source: Int J Mol Sci. 2025 Jan 20;26(2):859. doi: 10.3390/ijms26020859 (PMC11766212; doi:10.3390/ijms26020859)

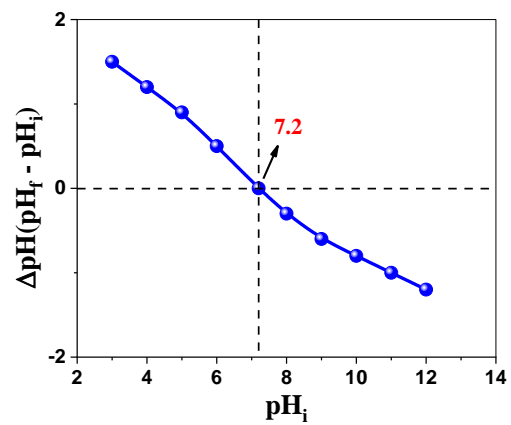

**Figure S1:** Point of Zero Charge of ZnO NPs

Supplement: Supplementary file 1 [file ijms-26-00859-s001.zip › ijms-3368262-supplementary.pdf]
